# B cell and monocyte phenotyping: A quick asset to investigate the immune status in patients with IgA nephropathy

**DOI:** 10.1371/journal.pone.0248056

**Published:** 2021-03-19

**Authors:** Senka Sendic, Ladan Mansouri, Sigrid Lundberg, Anna Nopp, Stefan H. Jacobson, Joachim Lundahl

**Affiliations:** 1 Division of Nephrology, Department of Clinical Sciences, Karolinska Institutet, Danderyd Hospital, Stockholm, Sweden; 2 Karolinska Institutet, Clinical Science and Education, Södersjukhuset, Stockholm, Sweden; Institut Cochin, FRANCE

## Abstract

**Background:**

IgA nephropathy (IgAN) advances from multiple pathogenic “hits” resulting in poorly O-galactosylated IgA1 glycoforms (Gd-IgA1), production of antibodies and glomerular deposition of immune complexes. A sequence of immune responses arising from plasma cells, T cells and antigen presenting cells (APCs), causes glomerular injury. This study was designed to phenotype subsets of B cells, monocytes and T cells in the peripheral circulation and their association with inflammatory cytokines and kidney function in patients with IgAN, healthy controls (HC) and disease controls with autosomal dominant polycystic kidney disease (ADPKD).

**Methods:**

Patients with IgAN (n = 13), median estimated glomerular filtration rate (eGFR) of 57 ml/min/1.73m^2^ (IQR 42–84), patients with ADPKD (n = 13) matched for kidney function, gender and age and gender and age-matched HC (n = 13) were recruited. CD3+ and CD3- peripheral blood mononuclear cells were isolated and profiled based on their specific surface markers for different subsets of monocytes, B and T cells and analyzed by flow cytometry. Cytokines were analyzed by ELISA.

**Results:**

We observed a significant decrease in the proportion of pre-switched B cells and plasmablasts, but an increase in long-lived plasma cells in the peripheral circulation of IgAN patients compared to HC. The proportion of non-classical monocytes was significantly higher in IgAN patients compared to both HC and ADPKD. We also report an association between sCD40L levels and the proportion of pre-switched B cells, as well as sCD40L and MCP-1 levels and albuminuria in IgAN patients.

**Conclusions:**

We applied an easy-access method to analyze subsets of immune cells as well as relevant inflammatory mediators in IgAN patients. Our data demonstrate an altered B cell profile that indicates a pathophysiological role of the B cell lineage and an increased proportion of non-classical monocytes that suggests their role in the disease process.

## Introduction

IgA nephropathy (IgAN) is the most common primary glomerulonephritis worldwide and is a leading cause of chronic kidney disease (CKD) and renal failure. Approximately 10–20% of patients reach end-stage renal disease (ESRD) 10 years after diagnosis [1–3].

Significant advances in understanding the pathogenesis of IgAN have been made in recent years. It is now widely accepted that IgAN does not arise from a single pathogenic “hit”, but rather arises due to multiple pathogenic “hits”, often referred to as the “multi-hit” hypothesis: an increased level of poorly O-galactosylated IgA1 glycoforms (Gd-IgA1) (Hit 1), production of O-glycan-specific antibodies (Hit 2), and the development of GdIgA1-containing immune complexes (GdIgA1-IC) (Hit 3). Deposition of GdIgA1-IC in the glomerular mesangium leads to mesangial cell proliferation and overproduction of extracellular matrix, cytokines and chemokines, culminating in glomerular injury (Hit 4) [4–7].

Currently, there is a consensus that aberrant IgA1 glycosylation is not able to induce renal injury on its own. A chain of immune responses, arising from plasma cells, T cells and antigen presenting cells (APCs) have an impact on disease outcomes. So far, the exact source of Gd-IgA1 during B cell maturation and trafficking is not fully delineated. Moreover, the level of crosstalk between relocated B cells and different T cell- and monocyte subsets needs further investigation.

Given the complex pathophysiology in IgAN, the interaction between different parts of the immune system needs to be further explored. Our approach was to apply a clinically feasible method using limited cell surface markers and minimal ex-vivo manipulation. We mapped the immune cells by phenotyping subsets of B cells, monocytes and T cells in the peripheral circulation of IgAN patients in comparison to those in healthy controls as well as in a disease control group. We also investigated the associations between cell subsets, inflammatory cytokines and renal function.

Our hypothesis was that an imbalance in the analyzed immunological network would disclose potential pathophysiological mechanisms.

## Material and methods

### Study population

In this cross-sectional study, 13 patients with biopsy verified IgAN and 13 patients with autosomal dominant polycystic kidney disease (ADPKD), as disease controls, were recruited. IgAN patients were enrolled between August 2017 and December 2017 and ADPKD patients between November and December 2018. All study participants were recruited from the Department of Nephrology at Danderyd University Hospital, Stockholm, Sweden, and submitted written informed consent. Patients planned for a routine visit to the outpatient clinic were screened for inclusion. In total 18 IgAN patients were included, two patients were excluded due to treatment with systemic corticosteroids and three patients declined to participate. The IgAN and ADPKD patients were matched for kidney function (estimated glomerular filtration rate, eGFR, ±10 ml/min/1.73 m^2^), gender and age (±10 years). Patients did not have any intercurrent illnesses and had stable treatment (i.e. no change in dose of angiotensin converting enzyme inhibitor (ACE-I) or angiotensin receptor blocker (ARB) within eight weeks from sampling). None of the IgAN patients had a history of macroscopic hematuria. Proteinuria was measured as urine albumin creatinine ratio (UACR). Exclusion criteria were ongoing treatment with systemic corticosteroids or other systemic immunosuppressive drugs, treatment with systemic corticosteroids within six months from sample draft, diabetes mellitus, neoplasms or other inflammatory diseases. Healthy controls (HC) (*n* = 13) were recruited by announcement and were gender- and age-matched (±5 years) with IgAN patients. Baseline characteristics of participants are presented in Table 1.

**Table 1:** Baseline characteristics of study subjects.

	Demographic characteristics of study objects					
	IgAN		ADPKD^1^		HC^2^	
	Median (IQR)	n (%)	Median (IQR)	n (%)	Median (IQR)	n (%)
**Age (years)**	45 (38–60)		42 (37–56)		44 (37–63)	
**Gender (men)**		6 (46.2%)		6 (46.2%)		6 (46.2%)
**eGFR**^3^ **(ml/min/1.73m**^**2**^**)**	57 (42–84)		57 (36–81)		81 (72–86)	
**SBP**^4^ **(mmHg)**	125 (112–136)		128 (120–142)		N/A	
**DBP**^5^ **(mmHg)**	80 (77–85)		83 (75–88)		N/A	
**ACEi**^6^ or **ARB**^7^		8 (61.5%)		9 (69.2%)	N/A	
**Lipid lowering drugs**		6 (46.2%)		4 (30.8%)	N/A	

^1^ADPKD: autosomal dominant polycystic kidney disease (disease controls),

^2^HC: Healthy controls,

^3^eGFR: estimated glomerular filtration rate,

^4^SBP: systolic blood pressure,

^5^DBP: diastolic blood pressure,

^6^ACEi: angiotensin converting enzyme inhibitor (enalapril),

^7^ARB: angiotensin receptor blocker (candesartan or valsartan),

N/A: not applicable. Values are given as median and interquartile range (25–75%).

### Cell preparation—Isolation of CD3+/CD3- immune cells

Fresh blood samples (30 mL) were drawn into EDTA tubes (Vacutainer, Becton Dickinson, UK) from patients, HC and ADPKD. Peripheral blood mononuclear cells (PBMCs) were isolated by density-gradient centrifugation (Ficoll-Paque PLUS; GE Healthcare, Sweden). Subsequently, CD3+ cells were isolated by positive selection using a magnetic cell-sorting system (Miltenyi Biotec GmbH, Germany) according to the manufacturer’s instruction. Following isolation, cells were divided into CD3+ cells (eluted from the columns) and CD3- cells (unlabeled cells). Cells were stained with Live/Dead Fixable Near-IR Dead cell stain kit (ThermoFisher Scientific Inc., USA) for 10 min for analysis of the live cell fraction. Subsequently, the CD3- cells were subjected to two panels stained for B cell- and monocyte surface markers.

The CD3+ PBMCs were subjected to surface marker staining for T cell subsets: CD4+/CD8+ CCR7+ CD45RA+ naïve T cell, CD4+/CD8+ CCR7+ CD45RA- central memory T cell, CD4+/CD8+ CCR7- CD45RA+ effector T cell, CD4+/CD8+ CCR7- CD45RA- effector memory T cell, CD4+ CXCR3+ CCR6- Th1 cell, CD4+ CXCR3- CCR6- Th2 cell, CD4+ CXCR3- CCR6+ Th17 cell and CD4+ CCR4+ CD25+ CD127^low^ regulatory T cells.

To phenotype B cells, cells were stained with anti-CD19, anti-CD27, anti-CD38 and anti-IgD (Biolegend Inc.) monoclonal antibodies. In the monocyte panel cells were stained with anti-CD14 and anti-CD16 (Biolegend Inc.) monoclonal antibodies. To phenotype T cell subsets, cells were stained with anti-CD4, anti-CD8, anti-CD45RA, anti-CCR7, anti-CXCR3, anti-CCR6, anti-CD25, anti-CD127 and anti-CCR4 (Biolegend Inc., UK. monoclonal antibodies Catalog number for antibodies are shown in S1 Table. These surface markers have earlier been suggested to identify different subsets of T-, B cells as well as monocytes in peripheral blood [8].

After 30 min staining, cells were washed and acquired on flow cytometry. Cells were analyzed within the gates on forward and side scatter plots and live cells were examined.

**Fig 1:**
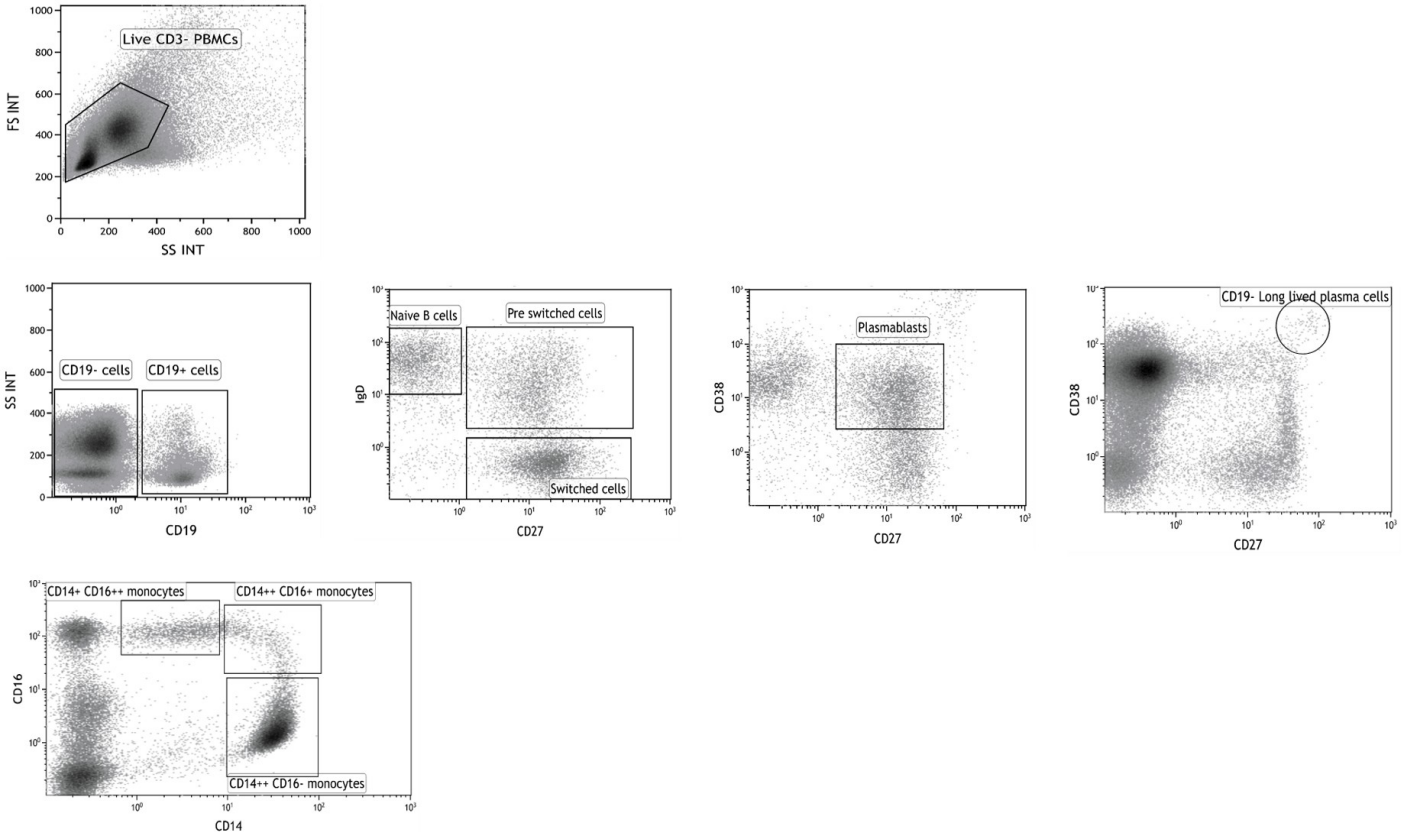
Gating strategies. Gating strategies for analysis of CD3- CD19+ CD27- IgD+ naïve B cells, CD3- CD19+ CD27+ IgD+ pre-switched B cells, CD3- CD19+ CD27+ IgD- switched B cells, CD3- CD19- CD27 ^hi^ CD38 ^hi^ long-lived plasma cells and monocyte subsets.

Gating strategies for analysis of B cell and monocyte subpopulations are shown in Fig 1.

### Analysis of cytokines with enzyme-linked immunosorbent assay

Plasma was obtained from whole blood, drawn in EDTA tube, by centrifuging at 400xg, for 10 min at room temperature. Plasma samples from IgAN patients and HC were then analyzed with enzyme-linked immunosorbent assay (ELISA) to determine the concentration of the following factors; monocyte chemoattractant protein (MCP-1) (Human CCL2/MCP-1 Quantikine ELISA Kit, R&D Systems, USA), soluble CD14 (Human CD14 Quantikine ELISA Kit, R&D Systems), soluble CD40L (Human CD40 Ligand/TNFSF5 Quantikine ELISA Kit, R&D Systems), B cell activating factor (BAFF) (Human BAFF Quantikine ELISA Kit, R&D Systems), IL-6 (Human IL-6 Quantikine HS ELISA Kit, R&D Systems), Fractalkine (Human CX3CL1/Fractalkine Quantikine ELISA Kit, R&D Systems) and MIP-1 (Human CCL3/MIP-1 alpha Quantikine ELISA Kit, R&D Systems). Catalog number for ELISA kits are shown in S2 Table. The samples were assayed in duplicates and the average values were considered for further analysis. The optical density (OD) in blank well was subtracted from the OD values in samples before calculation of concentrations.

### Statistical analysis

Scatter plots were prepared in GraphPad Prism 8 (GraphPad Software, Inc., USA). In scatter plots, whiskers represent 25–75% interquartile range (IQR) with the median shown by a line. Statistical analysis was done in GraphPad Prism 8 (GraphPad Software, Inc., USA), STATISTICA version 10 (StatSoft, Inc., USA) and IBM SPSS Statistics 25 (IBM Corp., USA). The cell fractions were compared between IgA patients, ADPKD and HC. The cytokine concentrations were compared between IgA patients and HC. Since the values were not normally distributed in all groups, comparisons for cell fractions were performed using the Kruskal-Wallis test and for cytokine concentrations Mann-Whitney U test was applied. Significant differences between groups were analyzed using the post hoc multiple comparisons p values (2-tailed) test. P<0.05 was considered statistically significant. Mann-Whitney U test was used to compare laboratory findings on blood parameters between study groups. Linear regression test was applied to determine the relationship between different variables; clinical indexes and cell fractions as well as cytokine levels.

### Statement of ethics

The study was approved by the Swedish Ethical Review Authority, Regional Ethical Committee and Institutional Review Board of the Karolinska Institutet, Stockholm, Sweden (2017/182-31/1). All study participants completed written informed consent.

## Results

### Laboratory findings and clinical data

There were no significant differences in blood pressure control and use of ACEi or ARB between IgAN and ADPKD groups (Table 1).

UACR was significantly higher and plasma albumin significantly lower in patients with IgAN compared to ADPKD (p<0.05, Table 2). There were no significant differences in CRP, Hb or WBC (Table 2).

**Table 2:** Laboratory data for blood parameters.

	Laboratory Findings		
	IgAN	ADPKD	P-value*
	Median (IQR)	Median (IQR)	
Creatinine (μmol/L)	88 (73–150)	99 (75–161)	0.626
UACR^1^ (mg/mmol)	74 (17.5–115.5)	1.6 (1–6.2)	0.001*
CRP^2^ (mg/L)	1 (1–1.5)	1 (1–4.5)	0.336
Hb^3^ (g/L)	136 (129–145)	129 (123–141)	0.248
WBC^4^ (10^9^/L)	5.9 (4.6–6.5)	6,2 (4.8–8.1)	0.293
P-Alb^5^ (g/L)	34 (31.5–37.0)	37 (35.5–40.0)	0.017*

^1^UACR: urine albumin creatinine ratio,

^2^CRP: c-reactive protein,

^3^Hb: Hemoglobin,

^4^WBC: white blood cell count,

^5^Alb: Albumin. Values are given as median and interquartile range (25–75%).

* Significant P-value, using Mann- Whitney U test.

### Flow cytometric analysis of CD3- (non T-lymphocyte), CD3+ (T- lymphocyte) and peripheral blood mononuclear cells (PBMCs)

#### B-lymphocyte subsets in IgAN

Based on the expression of the cell surface markers CD19, CD27, CD38 and IgD, the following B cell subsets were identified in the peripheral blood of patients with IgAN, ADPKD and HC: naïve B cells (CD19+ CD27-, IgD+), pre-switched B cells (CD19+ CD27+ IgD+), switched B cells (CD19+ CD27+ IgD-) plasmablasts (CD19+ CD27+ CD38+) and long-lived plasma cells (CD19-CD27^hi^ CD38^hi^).

**Fig 2:**
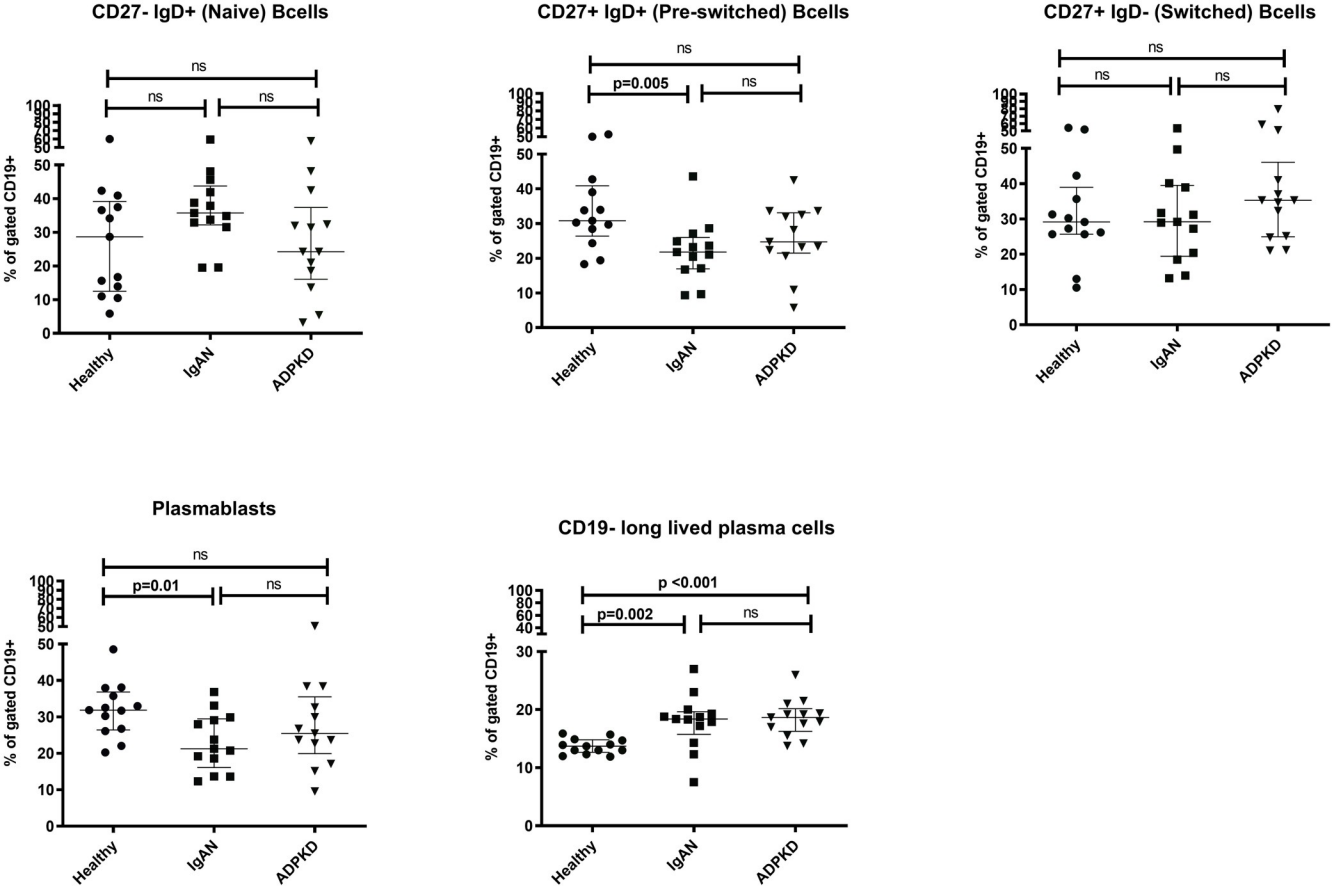
Proportions of B cell subsets. Proportions of B cell subsets in patients with IgA nephropathy (IgAN), autosomal dominant polycystic kidney disease (ADPKD) and in healthy controls (HC). Comparisons for cell fractions were performed using the Kruskal-Wallis test, P < 0.05 was considered statistically significant. Scatter plots represent the range with whiskers and the median as the middle line.

In the peripheral circulation of patients with IgAN, proportions of pre-switched B cells and plasmablasts were lower than those in HC (p = 0.005 and p = 0.01 respectively), while the proportion of CD19-negative long-lived plasma cells (LLPC) was higher (p = 0.02, Fig 2). There were no significant differences between patients with IgAN and HC comparing the proportions of naïve B cells and switched B cells. To assess if these differences are IgAN- specific, the cell fractions in IgAN patients were compared to those in ADPKD. A significant difference between the groups was observed for pre-switched B cells but not for plasmablasts or LLPC (Fig 2).

**Fig 3:**
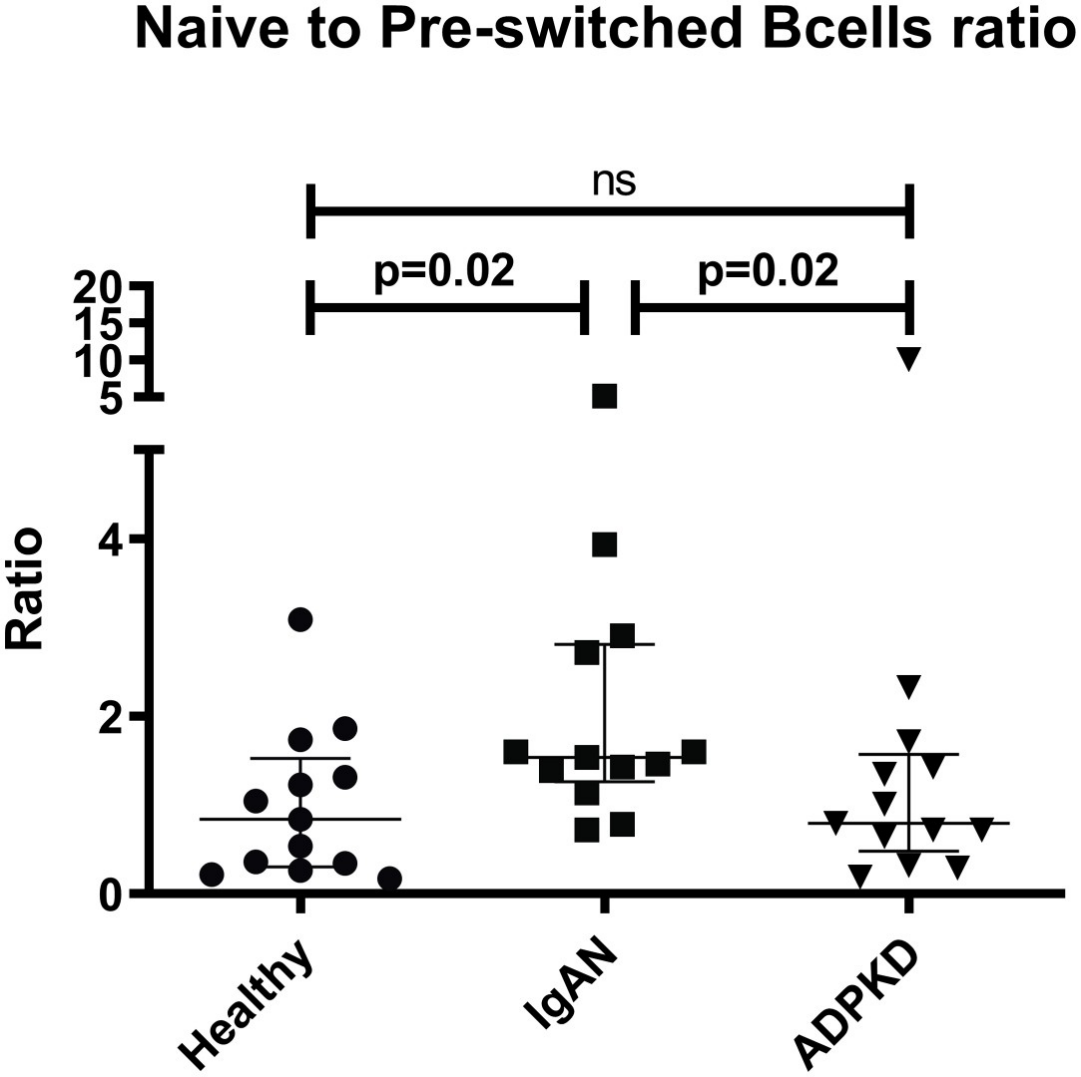
Ratio of naïve/pre-switched B cells. Ratio of naïve/pre-switched B cells in patients with IgA nephropathy (IgAN), autosomal dominant polycystic kidney disease (ADPKD) and healthy controls (HC). Comparisons for cell fractions were performed using the Kruskal-Wallis test, P < 0.05 was considered statistically significant. Scatter plots represent the range with whiskers and the median as the middle line.

Since we observed a similar proportion of naïve B cells in HC and IgAN patients, despite the differences in pre-switched B cells, we also analyzed the ratio between naïve and pre-switched B cells. The ratio between naïve and pre-switched B cell proportions was higher in IgAN patients compared to HC (p = 0.02) and ADPKD (p = 0.02) (Fig 3).

#### Monocyte subsets in IgAN

**Fig 4:**
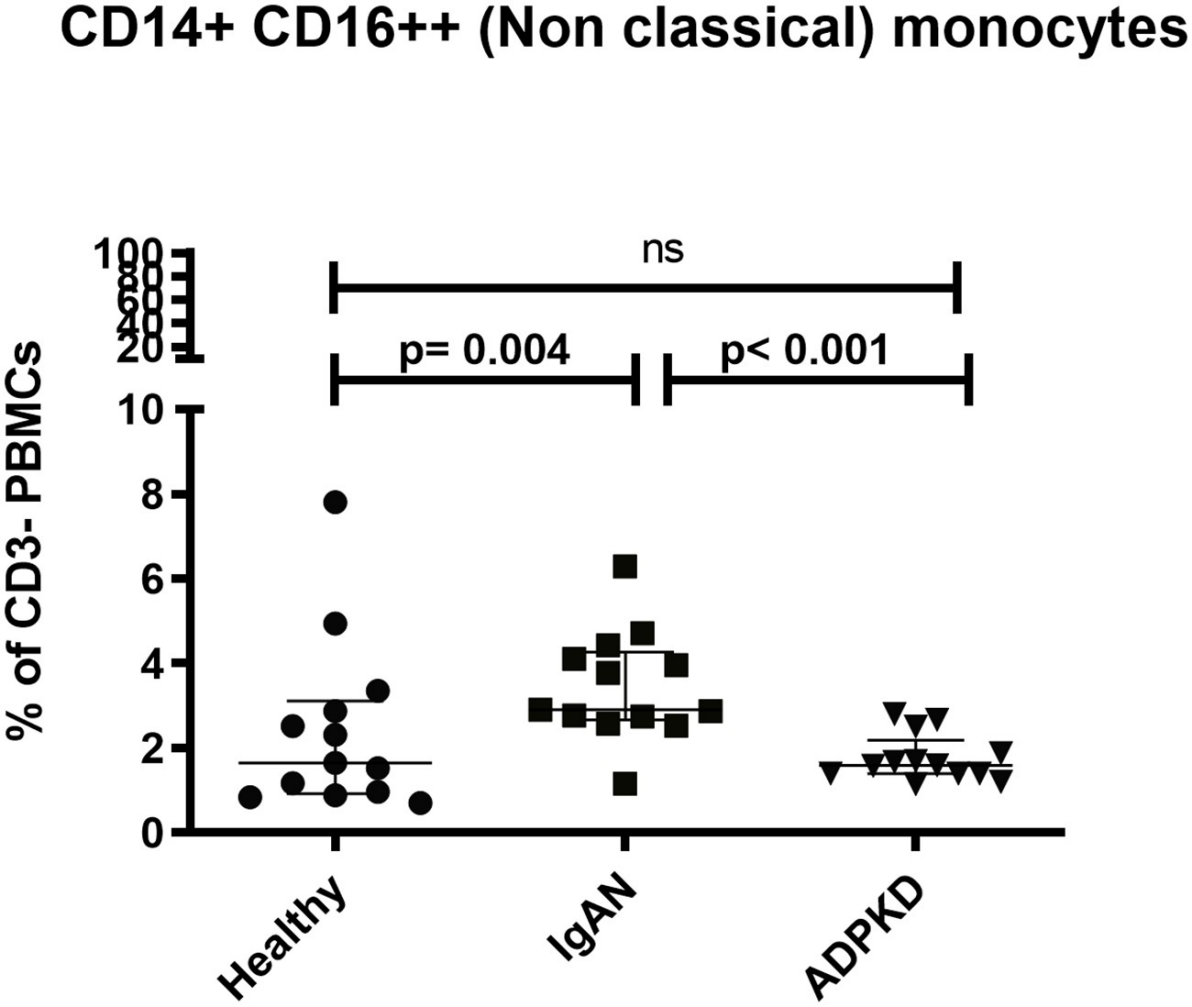
Proportions of non-classical monocytes. Proportions of non-classical monocytes in patients with IgAN, ADPKD and HC. Comparisons for cell fractions were performed using the Kruskal-Wallis test, P < 0.05 was considered statistically significant. Scatter plots represent the range with whiskers and the median as the middle line.

Based on the expression of the cell surface markers CD14 and CD16 we identified three monocyte subsets: classical monocytes (CD14++, CD16-), intermediate monocytes (CD14++ CD16+) and non-classical monocytes (CD14+ CD16++). Patients with IgAN had a higher proportion of non-classical monocytes (CD14+ CD16++) compared to both HC and patients with ADPKD (p = 0.004 and p< 0.001 respectively, (Fig 4). No significant differences were observed regarding classical monocyte- (CD14++, CD16-) or intermediate monocyte proportions (CD14++ CD16+) (S1 Fig).

#### T-lymphocyte subsets in patients with IgAN, disease controls and healthy controls

Comparing IgAN patients, ADPKD and HC, no significant differences were found in proportions of T-lymphocyte subsets; CD4+ naïve cells, CD4+ memory cells, CD8+ naïve cells or CD8+ memory cells (S3 Table.). No differences were detected in the fraction of T-helper cell subsets; Th1, Th2, Th17 as well as regulatory T cells (S4 Fig).

We also analyzed the ratios between proportions of pre-switched B cells and plasmablasts and Th2, as the cytokines produced by Th2 cells drive the differentiation and maturation of B cells. The ratios were significantly lower in IgAN patients compared to HC (p<0.001). The ADPKD also showed a lower pre-switched B cell/Th2 ratio compared to HC (p = 0.03), though this was not the case regarding plasmablast/Th2 ratio (S5 Fig).

### Characterization of cytokines in plasma by ELISA

**Fig 5:**
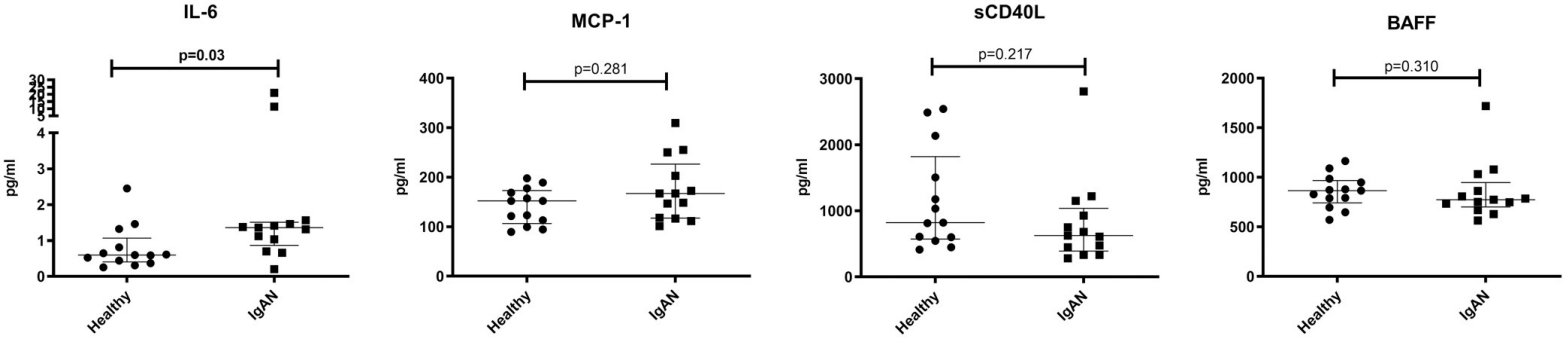
Cytokine levels. Levels of cytokines in plasma from IgAN patients and healthy controls. Mann-Whitney U test was used to compare cytokine concentrations, P < 0.05 was considered statistically significant. Scatter plots represent the range with whiskers and the median as the middle line.

We report significantly higher levels of IL-6 in IgAN patients compared to HC. However, we could not detect any significant differences in the levels of BAFF, CD40L or MCP-1 (Fig 5). No significant differences were detected in the levels of CD14, fractalkine or MIP-1 (S6 Fig).

### Relationship between clinical features, cytokine levels and immune cells

**Fig 6:**
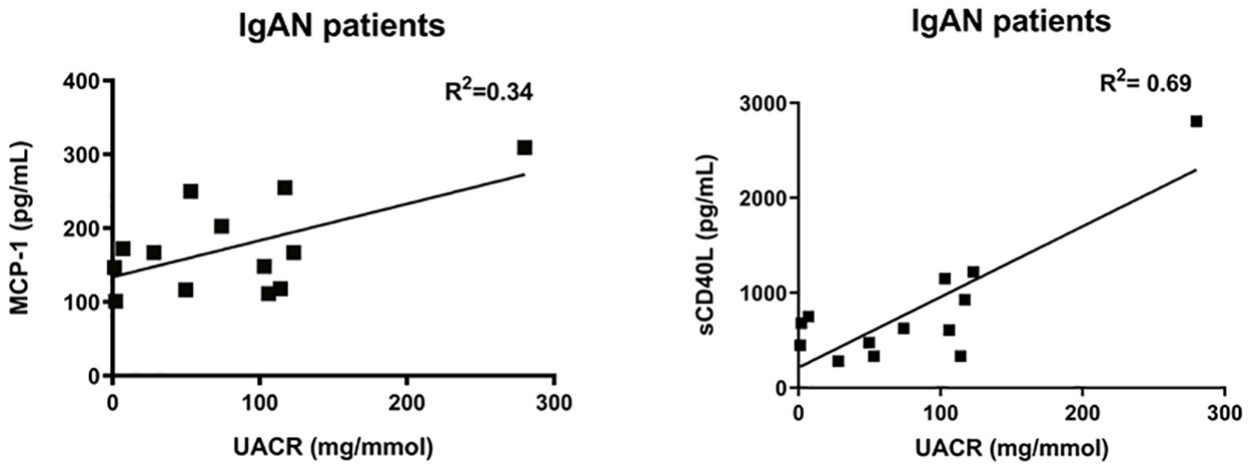
The relationship between cytokines and albuminuria. The relationship between MCP-1 / sCD40L levels and albuminuria in IgAN patients using linear regression test.

Linear regression test showed a correlation between MCP-1 levels (R^2^value = 0.34, p = 0.03) as well as sCD40L levels (R^2^ value = 0.69, p<0.001) and UACR in IgAN patients (Fig 6). We found no significant relationship between eGFR values or UACR and the proportions of B cells or monocytes in IgAN patients.

### Relationship between immune cells and cytokines

**Fig 7:**
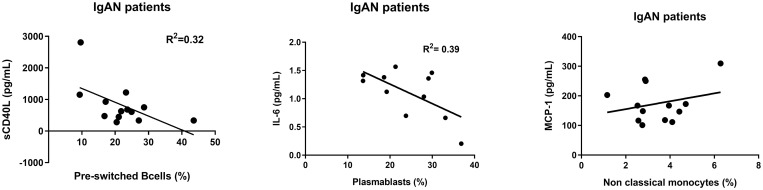
The relationship between cytokines and immune cell subtypes. The relationship between cytokines and immune cell subtypes in IgAN patients using linear regression test.

Levels of sCD40L showed a significant correlation (R^2^ value = 0.32, p = 0.04) with proportions of pre-switched B cells. Increasing proportions of pre-switched B cells were accompanied with decreasing amounts of sCD40L in IgAN, but not in HC (Fig 7). Levels of IL-6 showed a significant relationship (R^2^ value = 0.39, p = 0.03) with proportions of plasmablasts. However, there was no significant correlation between levels of MCP-1 and non-classical monocytes (R^2^ value = 0.07, p = 0.37) (Fig 7) or IL-6 levels and non-classical monocytes (S7 Fig).

## Discussion

In the present study we report differences in circulatory B cell subsets, monocyte subsets and IL-6 levels in IgAN patients, compared to healthy controls (HC) and/or disease controls i.e. patients with ADPKD. We also found associations between sCD40L levels and the proportion of pre-switched B cells, as well as sCD40L and MCP-1 levels and albuminuria in IgAN patients.

B cell development is an important subject to understand the pathophysiology of IgAN. IgD is expressed on naïve B cells before they are exposed to antigen and perform class-switching. IgD together with IgA are the only human immunoglobulins undergoing O-glycosylation [9, 10]. Changes in IgA O-glycosylation occur late during B cell maturation and imply production of Gd-IgA1 (Hit 1) and autoantibodies against Gd-IgA1 in IgAN (Hit 2) [4, 10], both events crucial in the pathological process [4, 11, 12]. Several immunological effector pathways are involved and orchestrate these events, for example T-lymphocytes, APCs and the cytokine/chemokine network. Given this immunological network, we aimed to simultaneously identify different B cell subsets together with T-cell, monocyte and cytokine/chemokine signatures with a method that minimize ex vivo manipulation.

We observed a significant lower proportion of pre-switched B cells and a higher trend in the naïve B cell population in IgAN compared to both HC and ADPKD. To validate these observations, we analyzed individual ratios between naïve and pre-switched B cells and disclosed an increased ratio in IgAN compared to HC and ADPKD. This may suggest an altered balance in B cell maturation in IgAN which could reflect a migration of pre-switched B cells from the circulation to for example follicles in the gut mucosa and Payer´s patches for further maturation and class-switching [13].

The proportions of plasmablasts in the peripheral circulation of IgAN patients were lower compared to HC. Plasma cells constitute an important B cell subset responsible for antibody production and their life-cycle in IgAN has been debated. Our data indicate a translocation of circulatory plasmablasts to lymphoid tissues for further maturation into antibody-producing cells. This assumption is supported by data that demonstrate migration of plasma cells to central lymphoid tissue, such as the bone marrow where they continue to produce underglycosylated antibodies [14, 15]. A lower number of polymeric IgA-secreting plasma cells in the duodenal mucosa but higher in systemic sites, for example in the bone marrow have been reported, which support a dynamic view of the cellular compartmentalize process in IgAN [4, 16]. A lower proportion of circulating plasmablasts in IgAN patients has also recently been reported by Cols et al. [17]. And their cohort was comparable to ours in terms of age and eGFR. However in the study by Si and co-workers the percentage of plasmablasts was elevated in IgAN [18]. In contrast their cohort included younger patients with more advanced disease compared to our patient group. This discrepancy indicate that age and clinical status are crucial parameters to consider when B cell subtype data are interpreted.

BAFF together with a proliferation inducing ligand (APRIL) are important factors in B cell development and class-switch and are of interest in the pathophysiology of IgAN as well as other autoimmune diseases [19]. Reports on BAFF levels in IgAN are however contradictory with some reports showing elevated BAFF levels in IgAN [20] while other reports indicate sustained levels of BAFF in IgAN compared to healthy controls [21]. Our results are in line with the latter showing no significant differences in the levels of BAFF (Fig 5).

Th2 cells play a major role in the orchestration of class-switching and maturation of B cells. We calculated the relationship between the plasmablasts and Th2 cells and noticed a lower ratio in IgAN which indicates a relative higher available circulating Th2 count per plasmablast. However, whether this relationship is valid also at the lymphoid tissue or whether it has any impact on the actual antibody production can only be speculated on. Overall, we found no significant differences in various subsets of T cells (S4 Fig) in IgAN compared to HC. T cells are believed to play an important role in IgAN [22]. He and co-workers found changes in T cell phenotype with decreased Th1/Th2 ratio in IgAN which was associated with proteinuria [23]. Increased percentages of Th2 and Th17 subpopulations were reported by Yang and co-workers [24] which is in consonance with the review from Ruszkowski and colleagues. In our study we noticed a trend towards increased levels of Th2 and Th17, although not reaching statistical significance (S4 Fig). The degree of proteinuria is one difference between the patients in our study compared to the cohort from He et al. (1 ±0.7 g/day) and Yang et al. (2.21 ±0.24 g/day), a condition that can be associated to changes in T cell phenotype (20).

We report a higher proportion of long-lived plasma cells (LLPC) in IgAN patients compared to HC. LLPC contribute to the humoral response by providing antibody production independent of remaining antibody producing plasma cells [25]. LLPCs consist partly of CD19-negative cells and reside mostly in bone marrow [25, 26] but can also be found in inflamed tissue such as in kidneys [26] or the intestinal mucosa [27]. To the best of our knowledge the CD19-negative LLPCs have not previously been studied in IgAN and it is currently not known in what way this subpopulation of B cells contributes to the pathological processes. An interesting finding is that polycystic kidney disease patients also had an expanded population of CD19-negative LLPC. A plausible explanation for this finding is not imminent but there is growing interest in how vascularization and lymphangiogenesis might affect cyst formation and growth, a process in which B cells are involved [28].

Our data showed an altered monocyte phenotype in patients with IgAN with an increased proportion of non-classical monocytes. Peripheral monocytes can be divided into three subgroups, classical (CD14++ CD16), intermediate (CD14++, CD16+) and non-classical (CD14+, CD16++) monocytes [29, 30] and our data go in line with a study by Cox and co-workers [31]. A noteworthy difference between our studies is that the patients had a more preserved renal function in the study by Cox and co-workers. An interpretation of this observation could be that changes in monocyte profile appear at an early stage of IgAN disease. In our study increased levels of non-classical monocytes were accompanied by elevated IL-6. Cox et al. did not analyze IL-6, and therefore we cannot further speculate whether an expanded population of non-classical monocytes precede an increase in IL-6.

CD16+ monocytes have previously gained attention and our group has reported increased CD16+ peripheral monocytes as well as their accumulation at the site of induced inflammation in patients with CKD [32] and Cols et al. reported a potential role of CD89^Low^ non-classical monocytes in the IgAN pathogenesis, which together set attention towards this monocyte subset [17]. Of interest is that we found a significant relationship between MCP-1 levels, a known chemotactic factor for monocytes, and albuminuria in the current study, which further indicate a role for these cells in the pathophysiology of IgAN. CD16+ monocytes are indeed of interest in other autoimmune diseases affecting the kidneys. Zhu et al. reported a pro-inflammatory role of these cells in systemic lupus erythematosus [33] and Burbano et al. reported a modulating role of CD16+ monocytes on classical monocytes [34]. In addition, Hotta and co-workers showed increased CD16+ monocytes in the peripheral circulation in patients with rapidly progressive crescentic glomerulonephritis, membranoproliferative glomerulonephritis, Henoch-Schönlein purpura nephritis and IgAN [35]. These results are in line with our findings. Since we have included a non-autoimmune kidney disease control group with comparable kidney function, our data support a role for non-classical monocytes in orchestrating the autoimmune component of IgAN.

We found a significant correlation between sCD40L levels and albuminuria and higher levels of sCD40L were related to a lower proportion of pre-switched B cells. This might indicate a role for the CD40-CD40L pathway in the IgAN pathophysiology, even though the exact in vivo mechanism of sCD40 is not fully understood. However, sCD40L has been suggested to act as a suppressor of the immune response by blocking the CD40-CD40L interaction between B- and T-cells, thereby affecting the B cell maturation and class-switch and inhibiting immunoglobulin production [36–38]. This is also supported by our findings of higher proportions of pre-switched, but not switched B cells in IgAN.

In this study we used a method that admits simultaneous analysis of several immune cells and soluble factors with a minimum of ex vivo manipulation. However, we used positive selection of CD3 cells which might possibly affect the cells, but the cells from IgAN and HC were treated in the same manner. In addition, cell staining was done according to The Human Immunology Project [8]. Since then our knowledge has expanded, e.g. among CD4+CXCR3-CCR6-cells some authors found other small cell subsets which are non-Th2 [39]. Hence, a different cell staining method might affect the results of T cell subsets. A limitation of our study is the lack of patients with macrohematuria which makes our results representative for IgAN patients without macrohematuria.

A further limitation of the study is the sample size and lack of sample size calculation. Even though the number of subjects is low, the study includes a well-defined group of IgAN patients, HC and a disease control group (ADPKD) to validate our results.

In conclusion, we applied an easy-access method to analyze subsets of immune cells as well as relevant inflammatory mediators in IgAN patients, healthy controls (HC) and disease controls with ADPKD. Our data demonstrate an altered B cell profile that indicates a pathophysiological role of the B cell linage in IgAN and an increased proportion of non-classical monocytes suggesting their role in the disease process.

## Supporting information

S1 Table Antibodies used in the study. (DOCX)

S2 Table ELISA-kit used in the study. (DOCX)

S3 Table Proportion of T-lymphocyte subsets in IgAN compared to healthy controls. (DOCX)

S4 Table Anonymized data clinical features and cytokines. (DOCX)

S5 Table Anonymized data cell subsets. (DOCX)

S1 Fig Proportions of different subsets of monocytes; CD14++CD16- (classical) and CD14++CD16+ (intermediate) in patients with IgA nephropathy, polycystic kidney disease and in healthy controls. (DOCX)

S2 Fig Gating strategies for analysis of T cells; a. Subsets of naïve and memory T cells. b. Subsets of T-helper cells. c. Regulatory T cells gated on CD3+CD4+CCR4+ CD25hi CD127lo cells d. Regulatory T cells gated on CD3+CD4+CD25hi CD127lo.
(DOCX)

S3 Fig Dot plot showing the differences in proportion of T-reg gated as: a) CD3+CD4+CCR4+CD25highCD127low, b) CD3+CD4+CD25+CD127low. (DOCX)

S4 Fig Comparison of T cell subsets’ fractions in patients with IgAN, ADPKD patients and healthy controls. (DOCX)

S5 Fig Comparison of pre switched B-cell—Th2 cell ratios and plasmablast-Th2 ratio between the groups. Ration between proportions of pre-switched B cells/Th2 cell. Comparisons for cell fractions were performed using the Kruskal-Wallis test, P < 0.05 was considered statistically significant. Scatter plots represent the range with whiskers and the median as the middle line.
(DOCX)

S6 Fig Levels of CD14, MIP-1 and fractalkine in plasma from IgA nephropathy patients and healthy controls. (DOCX)

S7 Fig The relationship between IL-6 and non-classical monocytes in IgAN patients using linear regression test. (DOCX)
