# Supplementary material for: B cell and monocyte phenotyping: A quick asset to investigate the immune status in patients with IgA nephropathy
Source: PLoS One. 2021 Mar 19;16(3):e0248056. doi: 10.1371/journal.pone.0248056 (PMC7978284; doi:10.1371/journal.pone.0248056)
Supplement: S1 Table — (DOCX) [file pone.0248056.s001.docx]

**S1 Table. Antibodies used in the study**

| Antibody | Supplier, catalog number |
| --- | --- |
| FITC anti-human CD4 Antibody | Biolegend Cat#317408 |
| PerCP/Cyanine5.5 anti-human CD8a Antibody | Biolegend Cat#300923 |
| APC anti-human CD197 (CCR7) Antibody | Biolegend Cat#353213 |
| PE anti-human CD45RA Antibody | Biolegend Cat# 304108 |
| APC anti-human CD196 (CCR6) Antibody | Biolegend Cat# 353415 |
| PerCP/Cyanine5.5 anti-human CD194 (CCR4) Antibody | Biolegend Cat# 359405 |
| PE anti-human CD25 Antibody | Biolegend Cat#302605 |
| APC anti-human CD127 (IL-7Rα) Antibody | Biolegend Cat#351315 |
| PerCP/Cyanine5.5 anti-human CD19 Antibody | Biolegend Cat#302229 |
| PE anti-human CD27 Antibody | Biolegend Cat# 302807 |
| APC anti-human CD38 Antibody | Biolegend Cat#356605 |
| FITC anti-human IgD Antibody | Biolegend Cat# 348205 |
| Alexa Fluor® 700 anti-human CD14 Antibody | Biolegend Cat# 367113 |
| PE/Cyanine7 anti-human CD16 Antibody | Biolegend Cat# 302015 |
