# Supplementary material for: B cell and monocyte phenotyping: A quick asset to investigate the immune status in patients with IgA nephropathy
Source: PLoS One. 2021 Mar 19;16(3):e0248056. doi: 10.1371/journal.pone.0248056 (PMC7978284; doi:10.1371/journal.pone.0248056)
Supplement: S2 Table — (DOCX) [file pone.0248056.s002.docx]

**S2 Table. ELISA-kit used in the study**

| ELISA | Supplier, catalog number |
| --- | --- |
| Human CCL2/MCP-1 Quantikine ELISA Kit | R&D Systems Cat#DCP00 |
| Human CD14 Quantikine ELISA Kit | R&D Systems Cat#DC140 |
| Human CD40 Ligand/TNFSF5 Quantikine ELISA Kit | R&D Systems Cat#DCDL40 |
| Human BAFF/BLyS/TNFSF13B Quantikine ELISA Kit | R&D Systems Cat# DBLYS0B |
| Human IL-6 Quantikine HS ELISA Kit | R&D Systems Cat#HS600B |
| Human CX3CL1/Fractalkine Quantikine ELISA Kit | R&D Systems Cat#DCX310 |
| Human CCL3/MIP-1 alpha Quantikine ELISA Kit | R&D Systems Cat#DMA00 |
