# Supplementary material for: B cell and monocyte phenotyping: A quick asset to investigate the immune status in patients with IgA nephropathy
Source: PLoS One. 2021 Mar 19;16(3):e0248056. doi: 10.1371/journal.pone.0248056 (PMC7978284; doi:10.1371/journal.pone.0248056)
Supplement: S3 Table — (DOCX) [file pone.0248056.s003.docx]

**S3 Table. Proportion of T-lymphocyte subsets in IgAN compared to healthy controls.**

|  | IgA Patients | Healthy Controls |  |
| --- | --- | --- | --- |
|  | Median (IQR% 25-75) | Median (IQR% 25-75) | P-value |
| CD4+ naïve T cells | 26 (23- 45.5) | 36 (29-42) | 0.36 |
| CD4+ memory T cells | 27(19- 34) | 28 (24- 34) | 0.54 |
| CD4+ effector T cells | 3(2- 16) | 2 (2-3) | 0.17 |
| CD4+ effector memory T cells | 34.5 (26-44) | 34 (25-39) | 0.61 |
| CD8+ naïve T cells | 24 (11.5-46) | 19 (10.5-35) | 0.54 |
| CD8+ memory T cells | 10 (3-14) | 10 (6-13) | 0.84 |
| CD8+ effector T cells | 41 (29-68) | 45 (24-57) | 0.54 |
| CD8+ effector memory T cells | 19 (10-23) | 23 (15-34) | 0.10 |
