# Supplementary material for: B cell and monocyte phenotyping: A quick asset to investigate the immune status in patients with IgA nephropathy
Source: PLoS One. 2021 Mar 19;16(3):e0248056. doi: 10.1371/journal.pone.0248056 (PMC7978284; doi:10.1371/journal.pone.0248056)
Supplement: S4 Table — (DOCX) [file pone.0248056.s004.docx]

**S4 Table. Anonymized data clinical features and cytokines.**

|  | eGFR ml/min | UACR mg/mmol | MCP1 pg/ml | sCD40L pg/ml | IL6 pg/ml | BAFF pg/ml | CD14 pg/ml | MIP-1 pg/ml | Fractalkine pg/ml |
| --- | --- | --- | --- | --- | --- | --- | --- | --- | --- |
| IgAN 1 | 88 | 7.1 | 172.3 | 751.1 | 1.3 | 668.9 | 1781.8 | 147.3 | 495.5 |
| IgAN 2 | 79 | 106 | 111.4 | 609.1 | 0.2 | 752.1 | 1756.9 | 153.3 | 540.3 |
| IgAN 3 | 83 | 117 | 255.2 | 928.5 | 1.4 | 863.0 | 1899.3 | 126.4 | 652.3 |
| IgAN 4 | 33 | 74 | 202.8 | 626.9 | 0.7 | 786.0 | 1967.2 | 123.4 | 921.0 |
| IgAN 5 | 30 | 53 | 250.2 | 334.1 | 1.0 | 749.0 | 1831.4 | 126.4 | 809.0 |
| IgAN 6 | 84 | 123 | 167.3 | 1221.3 | 21.5 | 1079.0 | 1829.8 | 126.2 | 741.9 |
| IgAN 7 | 57 | 1.3 | 147.0 | 449.5 | 1.6 | 564.2 | 1740.4 | 138.3 | 719.5 |
| IgAN 8 | 90 | 114 | 118.2 | 334.1 | 1.5 | 1032.4 | 2008.6 | 162.2 | 495.5 |
| IgAN 9 | 57 | 49.7 | 116.5 | 476.1 | 0.7 | 733.6 | 2096.3 | 150.3 | 809.0 |
| IgAN 10 | 75 | 2 | 101.3 | 680.1 | 1.4 | 773.7 | 1581.4 | 120.4 | 607.5 |
| IgAN 11 | 37 | 28 | 167.3 | 280.9 | 1.1 | 628.9 | 1884.4 | 120.2 | 764.3 |
| IgAN 12 | 49 | 103 | 148.6 | 1150.3 | 11.4 | 1719.3 | 2440.7 | 120.3 | 741.9 |
| IgAN 13 | 46 | 280 | 309.4 | 2809.3 | 1.4 | 807.5 | 1652.6 | 135.3 | 786.7 |
|  |  |  |  |  |  |  |  |  |  |
| Healthy 1 | 83 |  | 94.5 | 600.3 | 0.6 | 829.1 | 1843.0 | 132.4 | 876.3 |
| Healthy 2 | 85 |  | 113.1 | 1035.0 | 0.4 | 866.1 | 1930.8 | 135.4 | 741.9 |
| Healthy 3 | 86 |  | 99.6 | 609.1 | 0.2 | 872.2 | 2020.2 | 138.3 | 831.5 |
| Healthy 4 | 71 |  | 152.0 | 449.5 | 0.3 | 986.2 | 2237.1 | 153.3 | 607.5 |
| Healthy 5 | 70 |  | 121.6 | 547.0 | 0.8 | 792.1 | 1720.5 | 114.4 | 719.5 |
| Healthy 6 | 96 |  | 177.4 | 2135.0 | 0.6 | 949.2 | 1763.6 | 135.4 | 473.1 |
| Healthy 7 | 81 |  | 89.4 | 414.0 | 0.5 | 1164.8 | 2096.3 | 120.4 | 540.3 |
| Healthy 8 | 86 |  | 157.1 | 822.0 | 0.4 | 647.4 | 1560.0 | 126.4 | 585.0 |
| Healthy 9 | 69 |  | 197.7 | 1505.2 | 2.4 | 1090.9 | 1940.7 | 138.3 | 697.1 |
| Healthy 10 | 57 |  | 169.0 | 2490.0 | 1.3 | 789.1 | 1687.4 | 138.3 | 674.7 |
| Healthy 11 | 77 |  | 152.0 | 813.2 | 0.6 | 696.7 | 2202.3 | 153.3 | 831.5 |
| Healthy 12 | 88 |  | 189.3 | 1176.9 | 0.6 | 878.4 | 1382.7 | 168.2 | 652.3 |
| Healthy 13 | 73 |  | 123.3 | 2543.1 | 1.5 | 570.4 | 1692.4 | 147.3 | 562.7 |
|  |  |  |  |  |  |  |  |  |  |
|  |  |  |  |  |  |  |  |  |  |
| ADPKD 1 | 57 | 6.5 |  |  |  |  |  |  |  |
| ADPKD 2 | 82 | 1 |  |  |  |  |  |  |  |
| ADPKD 3 | 88 | 5.9 |  |  |  |  |  |  |  |
| ADPKD 4 | 40 | 1 |  |  |  |  |  |  |  |
| ADPKD 5 | 47 | 2.6 |  |  |  |  |  |  |  |
| ADPKD 6 | 80 | 0.7 |  |  |  |  |  |  |  |
| ADPKD 7 | 27 | 53 |  |  |  |  |  |  |  |
| ADPKD 8 | 89 | 1.6 |  |  |  |  |  |  |  |
| ADPKD 9 | 79 | 2.4 |  |  |  |  |  |  |  |
| ADPKD 10 | 44 | 1.3 |  |  |  |  |  |  |  |
| ADPKD 11 | 74 | 0.7 |  |  |  |  |  |  |  |
| ADPKD 12 | 31 | 1.2 |  |  |  |  |  |  |  |
| ADPKD 13 | 28 | 31.4 |  |  |  |  |  |  |  |
