# Supplementary material for: B cell and monocyte phenotyping: A quick asset to investigate the immune status in patients with IgA nephropathy
Source: PLoS One. 2021 Mar 19;16(3):e0248056. doi: 10.1371/journal.pone.0248056 (PMC7978284; doi:10.1371/journal.pone.0248056)
Supplement: S5 Table — (DOCX) [file pone.0248056.s005.docx]

**S5 Table. Anonymized data cell subsets.**

|  | CD4+CXCR3+CCR6- Th1 | CD4+CXCR3-CCR6-Th2 | CD4+CXCR3-CCR6+ Th17 | CD4+CXCR3+CCR6+ Th1 Th17 | CD4+CCR4+CD25+CD127low Treg | CD4+CD25+CD127low Treg |  | CD19+CD27-IgD+ Naive B | CD19+CD27+IgD+ Preswitched B | CD19+CD27+IgD- Switched B | CD19+CD27+CD38+Plasmablast | CD19-CD27+CD38+ Bcells |  | CD14++CD16- Classical M | CD14+ CD16+ Intermed. M | CD14+ CD16++ Nonclass M |
| --- | --- | --- | --- | --- | --- | --- | --- | --- | --- | --- | --- | --- | --- | --- | --- | --- |
| IgAN 1 | 12.6 | 14.0 | 4.2 | 8.7 | 9.3 | 5.1 |  | 41.9 | 28.7 | 13.2 | 13.6 | 27.0 |  | 33.8 | 2.0 | 4.7 |
| IgAN 2 | 14.9 | 14.34 | 6.3 | 13.7 | 7.2 | 2.7 |  | 19.5 | 24.9 | 49.7 | 36.9 | 17.9 |  | 42.7 | 2.9 | 4.1 |
| IgAN 3 | 18.8 | 26.7 | 9.1 | 19.0 | 4.4 | 1.8 |  | 19.5 | 17.1 | 53.6 | 18.6 | 20.0 |  | 32.9 | 1.5 | 2.9 |
| IgAN 4 | 13.6 | 7.0 | 10.5 | 35.2 | 10.7 | 4.3 |  | 34.8 | 21.8 | 38.9 | 33.1 | 23.0 |  | 46.5 | 3.0 | 1.2 |
| IgAN 5 | 13.5 | 31.9 | 2.9 | 6.3 | 14.1 | 4.2 |  | 31.5 | 43.6 | 18.5 | 28.0 | 18.3 |  | 49.9 | 4.3 | 2.9 |
| IgAN 6 | 9.9 | 8.5 | 9.5 | 16.7 | 6.9 | 3.0 |  | 35.7 | 23.2 | 29.2 | 20.8 | 18.7 |  | 33.7 | 3.0 | 2.5 |
| IgAN 7 | 12.0 | 23.3 | 2.6 | 13.0 | 7.0 | 3.1 |  | 33.8 | 21.1 | 31.7 | 21.3 | 17.2 |  | 19.7 | 2.6 | 4.4 |
| IgAN 8 | 12.9 | 18.0 | 7.0 | 16.8 | 5.3 | 2.5 |  | 38.8 | 27.1 | 28.9 | 29.9 | 18.4 |  | 28.6 | 1.8 | 3.8 |
| IgAN 9 | 16.0 | 5.5 | 9.6 | 40.2 | 6.3 | 3.3 |  | 45.6 | 16.8 | 27.2 | 23.8 | 18.8 |  | 19.4 | 2.9 | 2.6 |
| IgAN 10 | 11.9 | 4.5 | 13.2 | 32.9 | 4.7 | 3.9 |  | 32.9 | 23.6 | 31.2 | 29.1 | 12.3 |  | 40.9 | 1.6 | 2.7 |
| IgAN 11 | 7.9 | 7.9 | 13.0 | 34.2 | 5.4 | 3.5 |  | 59.4 | 20.4 | 14.0 | 19.2 | 14.3 |  | 23.2 | 2.0 | 3.9 |
| IgAN 12 | 15.9 | 11.0 | 9.9 | 29.0 | 3.1 | 2.4 |  | 48.1 | 9.3 | 20.4 | 12.3 | 19.3 |  | 43.0 | 2.9 | 2.8 |
| IgAN 13 | 20.9 | 7.3 | 8.3 | 31.1 | 10.0 | 5.5 |  | 37.9 | 9.6 | 40.1 | 13.6 | 7.5 |  | 40.9 | 2.2 | 6.3 |
|  |  |  |  |  |  |  |  |  |  |  |  |  |  |  |  |  |
| Healthy 1 | 9.9 | 16.2 | 7.0 | 24.4 | 5.8 | 3.3 |  | 11,0 | 50.1 | 31.3 | 30.3 | 14.0 |  | 35.4 | 3.2 | 2.5 |
| Healthy 2 | 15.0 | 8.9 | 10.2 | 22.2 | 6.8 | 3.2 |  | 16.7 | 30.8 | 42.3 | 38.1 | 13.9 |  | 27.0 | 0.9 | 1.2 |
| Healthy 3 | 22.8 | 5.3 | 7.5 | 30.5 | 8.0 | 3.6 |  | 42.3 | 24.3 | 25.7 | 26.7 | 13.7 |  | 24.5 | 0.7 | 0.7 |
| Healthy 4 | 19.0 | 10.3 | 8.7 | 23.7 | 6.1 | 3.2 |  | 36.6 | 29.7 | 27.3 | 26.1 | 11.9 |  | 12.7 | 1.2 | 0.9 |
| Healthy 5 | 16.5 | 5.0 | 9.5 | 40.3 | 8.8 | 3.7 |  | 37.5 | 28.4 | 25.7 | 31.7 | 13.0 |  | 37.8 | 1.6 | 1.5 |
| Healthy 6 | 10.6 | 4.5 | 12.5 | 37.0 | 7.6 | 4.4 |  | 10.5 | 30.3 | 52.0 | 32.5 | 15.7 |  | 38.8 | 2.5 | 3.3 |
| Healthy 7 | 20.0 | 6.1 | 7.6 | 25.6 | 7.0 | 5.1 |  | 5.8 | 33.8 | 54.3 | 35.7 | 15.9 |  | 39.0 | 3.7 | 7.8 |
| Healthy 8 | 25.8 | 7.0 | 6.2 | 20.7 | 13.4 | 7.2 |  | 13.9 | 52.7 | 26.2 | 31.9 | 13.0 |  | 17.3 | 0.2 | 0.8 |
| Healthy 9 | 13.8 | 8.0 | 9.8 | 30.0 | 7.8 | 4.6 |  | 34.1 | 18.3 | 30.2 | 22.1 | 14.7 |  | 23.5 | 2.6 | 4.9 |
| Healthy 10 | 14.6 | 10.4 | 10.5 | 22.5 | 7.0 | 4.3 |  | 40.9 | 39.0 | 13.0 | 33.0 | 13.0 |  | 32.2 | 2.2 | 1.6 |
| Healthy 11 | 9.0 | 11.4 | 5.9 | 10.7 | 10.5 | 6.1 |  | 60,0 | 19.4 | 10.5 | 20.2 | 12.3 |  | 27.7 | 2.0 | 2.3 |
| Healthy 12 | 11.5 | 7.4 | 8.5 | 26.1 | 7.0 | 3.3 |  | 28.6 | 33.9 | 29.1 | 48.5 | 12.0 |  | 44.7 | 3.2 | 2.9 |
| Healthy 13 | 16.8 | 8.9 | 8.5 | 34.0 | 6.2 | 3.9 |  | 15.6 | 42.7 | 35.7 | 37.9 | 14.9 |  | 47.0 | 2.0 | 1.0 |
|  |  |  |  |  |  |  |  |  |  |  |  |  |  |  |  |  |
|  |  |  |  |  |  |  |  |  |  |  |  |  |  |  |  |  |
| ADPKD 1 | 19.8 | 4.5 | 10.9 | 46.8 | 3.3 | 2.0 |  | 5.4 | 28.2 | 58.5 | 50.7 | 13.8 |  | 38.5 | 1.4 | 1.7 |
| ADPKD 2 | 10.6 | 10.5 | 5.19 | 8.3 | 5.3 | 2.3 |  | 48.1 | 20.7 | 24.8 | 23.7 | 19.3 |  | 20.5 | 0.9 | 1.9 |
| ADPKD 3 | 17.1 | 8.5 | 6.3 | 14.8 | 7.0 | 3.3 |  | 18.6 | 23.4 | 51.1 | 32.6 | 15.5 |  | 29.4 | 1.3 | 1.6 |
| ADPKD 4 | 13 | 7.5 | 14.4 | 24.4 | 8.8 | 4.0 |  | 31.9 | 22.4 | 34.6 | 22.7 | 14.2 |  | 34.3 | 2.1 | 1.1 |
| ADPKD 5 | 19.4 | 19.6 | 3.5 | 6.7 | 15.5 | 5.6 |  | 57.7 | 5.7 | 21.0 | 9.6 | 26.0 |  | 35.6 | 2.6 | 1.6 |
| ADPKD 6 | 19.1 | 15.2 | 8.0 | 23.0 | 7.7 | 3.2 |  | 21,0 | 32.5 | 37.0 | 29.9 | 17.6 |  | 60.0 | 3.4 | 2.7 |
| ADPKD 7 | 9.49 | 8.3 | 11.1 | 26.2 | 7.8 | 4.3 |  | 13.6 | 42.5 | 32.3 | 23.7 | 17.9 |  | 51.1 | 1.8 | 1.2 |
| ADPKD 8 | 13 | 6.5 | 7.5 | 20.5 | 5.2 | 2.4 |  | 42.4 | 24.7 | 25.1 | 26.8 | 18.6 |  | 38.7 | 2.2 | 1.4 |
| ADPKD 9 | 10.7 | 9.2 | 6.3 | 12.4 | 5.8 | 4.4 |  | 24.2 | 33.6 | 35.3 | 38.4 | 19.2 |  | 53.3 | 2.5 | 2.8 |
| ADPKD 10 | 26.3 | 12.0 | 6.2 | 15.8 | 8.8 | 5.6 |  | 32.4 | 32.0 | 21.2 | 15.2 | 21.5 |  | 41.5 | 2.0 | 1.7 |
| ADPKD 11 | 9.2 | 19.0 | 9.9 | 9.8 | 14.3 | 7.4 |  | 31.4 | 23.2 | 41.0 | 17.1 | 21.0 |  | 26.6 | 0.5 | 1.4 |
| ADPKD 12 | 14.6 | 9.5 | 13.4 | 31.8 | 12.2 | 7.3 |  | 3.2 | 10.9 | 79.6 | 25.5 | 17.0 |  | 47.0 | 3.0 | 2.5 |
| ADPKD 13 | 10.0 | 11.1 | 12.2 | 23.7 | 10.0 | 5.6 |  | 24.2 | 33.6 | 35.3 | 38.4 | 19.2 |  | 39.5 | 1.4 | 1.4 |
