# Supplementary material for: B cell and monocyte phenotyping: A quick asset to investigate the immune status in patients with IgA nephropathy
Source: PLoS One. 2021 Mar 19;16(3):e0248056. doi: 10.1371/journal.pone.0248056 (PMC7978284; doi:10.1371/journal.pone.0248056)
Supplement: S1 Fig — (DOCX) [file pone.0248056.s006.docx]

**S1 Fig. Proportions of different subsets of monocytes; CD14++CD16- (Classical) and CD14++CD16+ (intermediate) in patients with IgA nephropathy, polycystic kidney disease and in healthy controls**

**
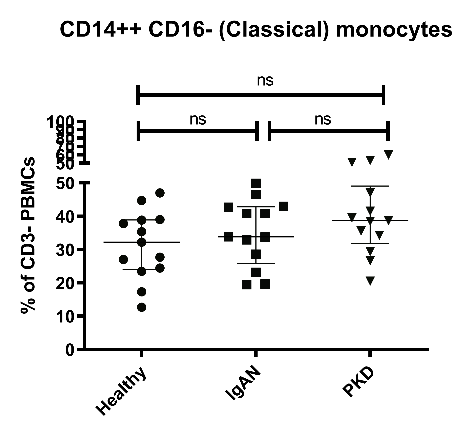

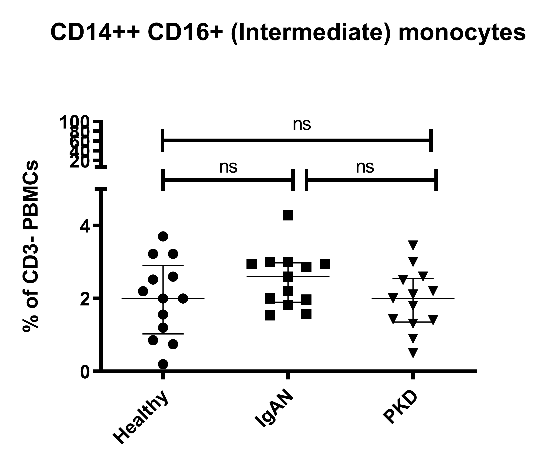
**
