# Supplementary material for: B cell and monocyte phenotyping: A quick asset to investigate the immune status in patients with IgA nephropathy
Source: PLoS One. 2021 Mar 19;16(3):e0248056. doi: 10.1371/journal.pone.0248056 (PMC7978284; doi:10.1371/journal.pone.0248056)

**S2 Fig. Gating strategies for analysis of T cells; a. Subsets of naïve and memory T cells. b. Subsets of T-helper cells. c. Regulatory T cells gated on CD3+CD4+CCR4+ CD25hi CD127lo cells d. Regulatory T cells gated on CD3+CD4+CD25hi CD127lo**

a.


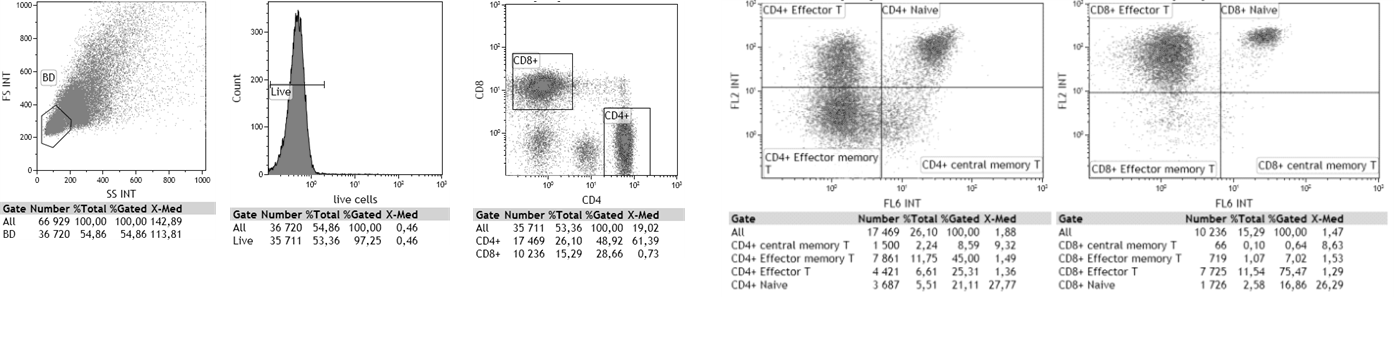


b.


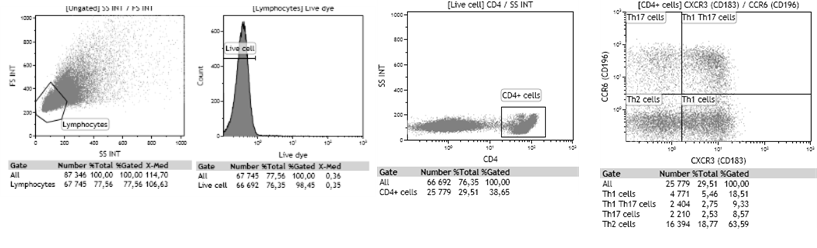


c.


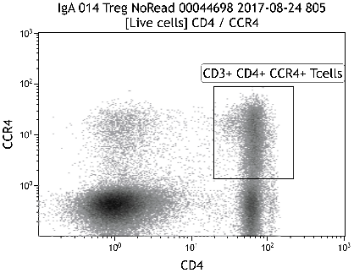

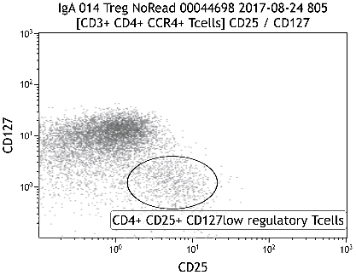


d.


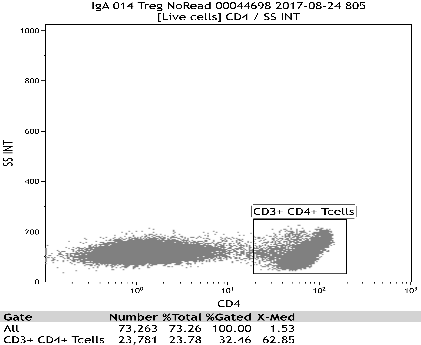

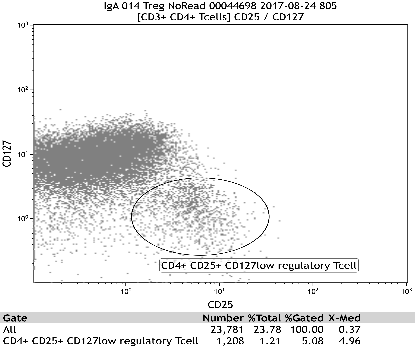

Supplement: S2 Fig — Gating strategies for analysis of T cells; a. Subsets of naïve and memory T cells. b. Subsets of T-helper cells. c. Regulatory T cells gated on CD3+CD4+CCR4+ CD25hi CD127lo cells d. Regulatory T cells gated on CD3+CD4+CD25hi CD127lo. (DOCX) [file pone.0248056.s007.docx]
