# Supplementary material for: B cell and monocyte phenotyping: A quick asset to investigate the immune status in patients with IgA nephropathy
Source: PLoS One. 2021 Mar 19;16(3):e0248056. doi: 10.1371/journal.pone.0248056 (PMC7978284; doi:10.1371/journal.pone.0248056)
Supplement: S3 Fig — (DOCX) [file pone.0248056.s008.docx]

**S3 Fig. Dot plot showing the differences in proportion of T-reg gated as: a) CD3+CD4+CCR4+CD25highCD127low, b) CD3+CD4+CD25+CD127low**

1. b)
