# Supplementary material for: B cell and monocyte phenotyping: A quick asset to investigate the immune status in patients with IgA nephropathy
Source: PLoS One. 2021 Mar 19;16(3):e0248056. doi: 10.1371/journal.pone.0248056 (PMC7978284; doi:10.1371/journal.pone.0248056)
Supplement: S5 Fig — Ration between proportions of pre-switched B cells/Th2 cell. Comparisons for cell fractions were performed using the Kruskal-Wallis test, P < 0.05 was considered statistically significant. Scatter plots represent the range with whiskers and the median as the middle line. (DOCX) [file pone.0248056.s010.docx]

**S5 Fig. Comparison of pre switched B-cell - Th2 cell ratios and plasmablast-Th2 ratio between the groups**


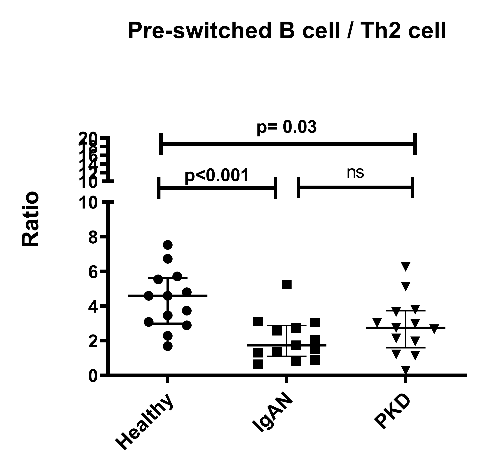

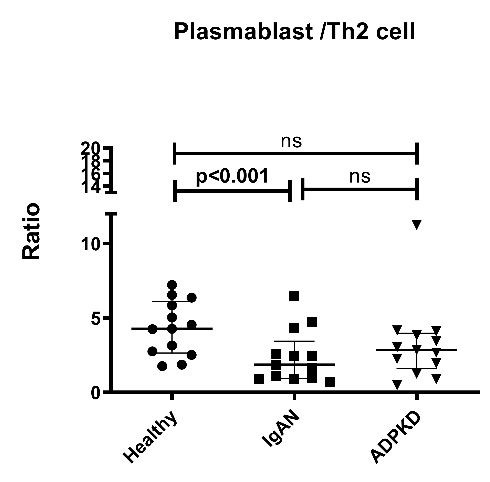


**Comparison of pre switched B-cell - Th2 cell ratios between the groups.** Ration between proportions of pre-switched B cells/Th2 cell. Comparisons for cell fractions were performed using the Kruskal-Wallis test, P < 0.05 was considered statistically significant. Scatter plots represent the range with whiskers and the median as the middle line.
