# Supplementary material for: B cell and monocyte phenotyping: A quick asset to investigate the immune status in patients with IgA nephropathy
Source: PLoS One. 2021 Mar 19;16(3):e0248056. doi: 10.1371/journal.pone.0248056 (PMC7978284; doi:10.1371/journal.pone.0248056)

**S6 Fig. Levels of CD14, MIP-1 and fractalkine in plasma from IgA nephropathy patients and healthy controls**


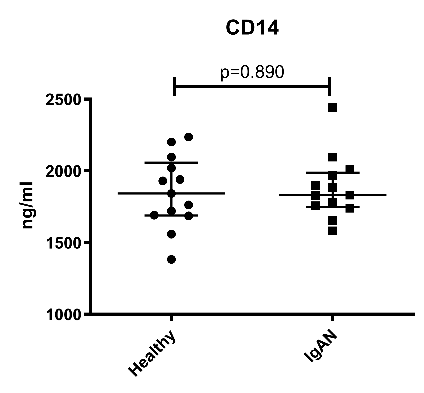

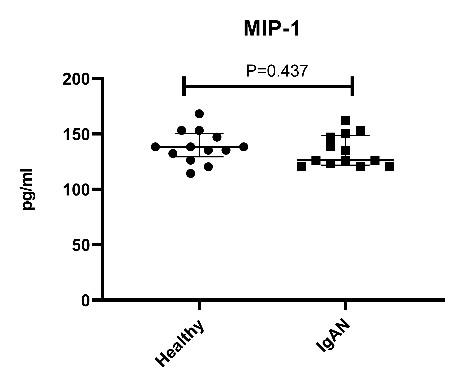

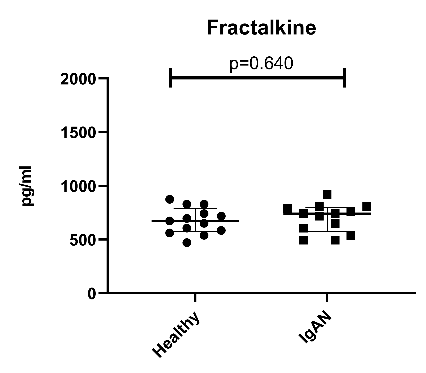

Supplement: S6 Fig — (DOCX) [file pone.0248056.s011.docx]
