# Supplementary material for: B cell and monocyte phenotyping: A quick asset to investigate the immune status in patients with IgA nephropathy
Source: PLoS One. 2021 Mar 19;16(3):e0248056. doi: 10.1371/journal.pone.0248056 (PMC7978284; doi:10.1371/journal.pone.0248056)
Supplement: S7 Fig — (DOCX) [file pone.0248056.s012.docx]

**S7 Fig. The relationship between IL-6 and non-classical monocytes in IgAN patients using linear regression test.**
